# Distinct metabolic signatures in blood plasma of bisphenol A–exposed women with polycystic ovarian syndrome

**DOI:** 10.1007/s11356-023-26820-w

**Published:** 2023-04-15

**Authors:** Navya B. Prabhu, Sampara Vasishta, Shashikala K. Bhat, Manjunath B. Joshi, Shama Prasada Kabekkodu, Kapaettu Satyamoorthy, Padmalatha S. Rai

**Affiliations:** 1 Department of Biotechnology, Manipal School of Life Sciences, Manipal Academy of Higher Education, Manipal, 576104 India; 2 Department of Ageing Research, Manipal School of Life Sciences, Manipal Academy of Higher Education, Manipal, 576104 India; 3 Department of Obstetrics and Gynaecology, Dr. TMA Pai Hospital, Melaka Manipal Medical College, Manipal Academy of Higher Education, Manipal, 576101 India; 4 Department of Cell and Molecular Biology, Manipal School of Life Sciences, Manipal Academy of Higher Education, Manipal, 576104 India

**Keywords:** Polycystic ovarian syndrome, Bisphenol A, Metabolites, Metabolic pathway, Sphingolipids, Steroids

## Abstract

Polycystic ovarian syndrome (PCOS) is a complicated endocrinopathy with an unclear etiology that afflicts fertility status in women. Although the underlying causes and pathophysiology of PCOS are not completely understood, it is suspected to be driven by environmental factors as well as genetic and epigenetic factors. Bisphenol A (BPA) is a weak estrogenic endocrine disruptor known to cause adverse reproductive outcomes in women. A growing relevance supports the notion that BPA may contribute to PCOS pathogenesis. Due to the indeterminate molecular mechanisms of BPA in PCOS endocrinopathy, we sought liquid chromatography with tandem mass spectrometry (LC–MS/MS), a metabolomics strategy that could generate a metabolic signature based on urinary BPA levels of PCOS and healthy individuals. Towards this, we examined urinary BPA levels in PCOS and healthy women by ELISA and performed univariate and chemometric analysis to distinguish metabolic patterns among high and low BPA in PCOS and healthy females, followed by pathway and biomarker analysis employing MetaboAnalyst 5.0. Our findings indicated aberrant levels of certain steroids, sphingolipids, and others, implying considerable disturbances in steroid hormone biosynthesis, linoleic, linolenic, sphingolipid metabolism, and various other pathways across target groups in comparison to healthy women with low BPA levels. Collectively, our findings provide insight into metabolic signatures of BPA-exposed PCOS women, which can potentially improve management strategies and precision medicine.

**Supplementary Information:**

The online version contains supplementary material available at 10.1007/s11356-023-26820-w.

## Introduction

Polycystic ovarian syndrome (PCOS) is a most prevalent and complex endocrinopathy marked by ovulatory dysfunction and abnormalities in insulin secretion, androgen synthesis, and relative gonadotropin ratios which generally lead to infertility in females. Women suffering from PCOS are more likely to develop hyperglycaemia, hypertension, type 2 diabetes mellitus, hyperlipidaemia, cardiovascular disease, and eventually develop metabolic syndrome in later stages (Prabhu et al. 2022; Sagvekar et al. 2018). Although the exact pathophysiology of this complicated lifestyle disorder is undetermined, it is believed to be caused by a complex interplay between different factors including ethnicity, genetic and epigenetic predisposition, and environmental facets (Prabhu et al. 2021). Environmental influences such as endocrine-disrupting chemicals (EDCs) may increase symptoms of PCOS or promote the ultimate PCOS phenotype in genetically susceptible women (Lazúrová et al. 2021). Analysis of blood and body fluids confirms almost all humans have a body burden of EDCs such as bisphenol A (BPA), parabens, phthalates, and polychlorinated biphenyls (Gore et al. 2014). These chemicals cause disturbances in the reproductive system due to their imitative action on endogenous hormones (Barrett and Sobolewski 2014).

BPA is one of the most abundantly available EDC, serves as a precursor for polycarbonates, polyesters, polysulfones, polyether ketones, and a major class of epoxy resins. It has a wide range of applications, and is commonly found in baby bottles, linings for metal-based food and plastic cans, optical lenses, electric equipment, plastic water ducts, dental sealing, and billing receipts (Rutkowska and Rachoń 2014). Although the potential means of exposure to BPA in humans are inhalation, ingestion, and dermal uptake, few studies reported that BPA exposure solely occurs through dietary consumption (Ohore and Zhang 2019; Mukhopadhyay et al. 2022).

Human studies reported that BPA levels are substantially elevated in body fluids of PCOS women compared to healthy females, but the clinical significance as well as causality is yet unknown (Lazúrová et al. 2021). Additionally, it was perceived that serum BPA levels are relatively higher in females suffering from ovarian dysfunction and obesity and are in positive association with androgens, implying that BPA may enhance or suppress androgen metabolism and action in the liver (Takeuchi et al. 2004). Nevertheless, given that females with PCOS have greater circulating androgens than healthy females, and that higher androgen levels reduce BPA elimination. Henceforth, researchers hypothesized that excessive BPA may possibly signify a consequence rather than a cause (Fernández et al. 2010). In addition, recent evidence suggests that BPA may decrease ovarian steroidogenesis, leading to a contentious explanation of the link between BPA and ovarian steroids (Lazúrová et al. 2021).

Modern research is increasingly concentrating on the identification of potential biomarkers to comprehend the metabolic abnormalities in PCOS (Vonica et al. 2020). The assessment of relative amounts of metabolites, which may be affected by disorders, pathogens, and exposure to substances in the environment, is accomplished through metabolomics (Cho et al. 2018). Plasma metabolomics provides thorough monitoring of small-molecule degradation products downstream of gene and protein expression, allowing researchers to uncover key metabolic processes linked to PCOS (Chang et al. 2017).

A large number of studies have identified various metabolites involved in BPA metabolism and PCOS pathogenesis distinctly, but the impact of BPA in PCOS pathogenesis has not been reported. Henceforth, we employed LC–MS/MS to assess the alteration in metabolites and their patterns in PCOS and healthy control women. The current study may increase the probabilities of detecting possible metabolites which can lead to a better understanding of the multifarious role of PCOS and highlight the risk posed by exposure to BPA in PCOS and healthy females.

## Materials and methods

### Study participants and sample collection

**Fig. 1:**
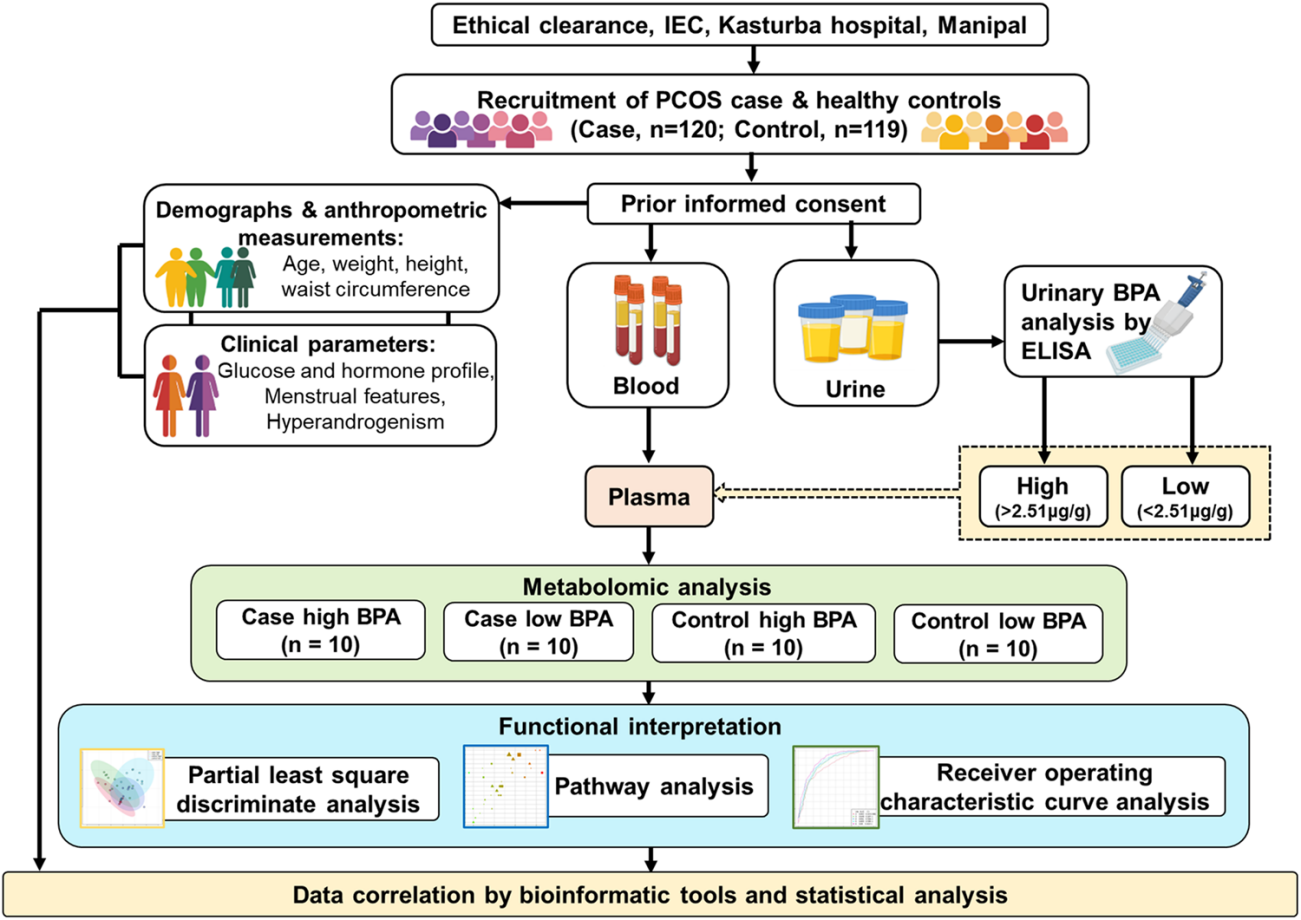
A comprehensive workflow of the present investigation

Ethical clearance for the study protocol was obtained from Institutional Ethics Committee, Kasturba Hospital, Manipal (IEC 366/2018). After obtaining approval, clinically diagnosed PCOS patients and healthy control participants were enrolled for the study at the Department of Obstetrics and Gynaecology, Dr. TMA Pai Hospital, Udupi. Patients who come to the hospital for a check-up and then met the inclusion criteria for the study were asked to participate voluntarily. Women between the age group 18 and 45 diagnosed with PCOS according to Rotterdam diagnostic criteria (2003) (Teede et al. 2018) were included in the study. Females who have not attained menarche, have attained menopause, and patients diagnosed with chronic illness except diabetes mellitus and cardiovascular disease were excluded from the study. Regularly menstruating women without any clinical complications were considered for the control group after a pelvic ultrasound examination. Based on the inclusion and exclusion criteria, the participants were classified into PCOS cases and controls. After taking informed consent from participants, 3 mL of peripheral blood and 10 mL of urine samples were collected. The comprehensive workflow of the present investigation is portrayed in Fig. 1.

### Clinical parameters of the study participants

The clinical variables such as blood pressure, body mass index, and homeostasis model assessment–estimated insulin resistance (HOMA IR); glucose profile including random blood sugar (RBS), fasting blood sugar (FBS), and glycated hemoglobin (HbA1c); hormone profile including follicle-stimulating hormone (FSH), luteinizing hormone (LH), prolactin, anti-mullerian hormone (AMH), and thyroid stimulating hormone (TSH); menstrual features including menarche and presence of menstrual cramps; clinical hyperandrogenism including acne, alopecia, and modified Ferriman-Gallwey score (mFG) were tabulated from the hospital reports.

### Estimation of urinary BPA in healthy control and PCOS women

The urine samples stored at − 80 °C were thawed and used for the estimation of BPA by ELISA kit as per the manufacturer’s instructions (Creative diagnostics, USA) (Prabhu et al. 2022) and the concentration of BPA was adjusted with creatinine. A duplicate evaluation of each test sample and standard was conducted.

### Sample preparation and LC–MS instrument settings

The plasma samples stored at − 80 °C were used for the metabolite extraction using chilled methanol. We have segregated the plasma samples of the participants into 4 groups with case high group having a high level of BPA in the PCOS females (case high), case low group having a low level of BPA in the PCOS women (case low), control high group having a high level of BPA in the control women (control high), and control low group having a low level of BPA in the control women (control low); each group with ten samples was categorized based on the threshold of 2.51 μg/g BPA in the urine (Zhang et al. 2011).

One hundred microliters of plasma samples was mixed with double the volume of chilled methanol and subjected to centrifugation for 15 min at 12,000 rpm. The obtained extract was dehydrated using vacuum CentriVap vacuum concentrator (Labconco, Kansas City, MO, USA) and deposits were reconstituted in 30 µL of water:acetonitrile (95:5) having 0.1% formic acid. All the samples were run in triplicates in ESI-positive mode. Solvent (A) contains 0.1% formic acid in HPLC-grade water, while solvent (B) contains 0.1% formic acid in 90% acetonitrile. For 45 min, an analytical C18 column (4.6150 mm, 3.5 microns) was employed at a flow rate of 0.4 mL/min. 0.0–1.5 min 92% A and 8% B, 1.5–25.0 min 92% A and 8% B, 25.0–35.0 min 2% A and 98% B, 35.0–35.5 min 2% A and 98% B, and 35.5–45 min 92% A and 8% B were the gradient conditions employed in the experiment. The capillary’s voltage was set to 3500 V. The drying gas temperature and flow rate were 250 °C and 8 L/min, respectively. Similarly, MS/MS was conducted to determine the MS feature fragmentation patterns. The source parameters were same as LC–MS when the auto MS/MS option was used.

### Metabolic profiling and data analysis

The obtained LC–MS data were processed in Metaboanalyst 5.0 (Pang et al. 2021) using the following adducts: single-charge proton, ammonium, methyl, sodium, potassium, adducts in positive mode using a tolerance limit of ± 15 ppm to improve sensitivity while minimizing background compounds. Univariate and multivariate analyses were performed to detect molecular features that differentiated PCOS and control women with high and low BPA levels. The software tool MSConvert (Adusumilli and Mallick 2017) was used to convert raw spectrometric data (.d files) to mzML format. Using MetaboAnalyst 5.0, the data were normalized further processed by log transformation and Pareto scaling, to acquire uniform individual characteristics before conducting statistical analysis. Additionally, partial least squares-discriminant analysis (PLS-DA) (supervised multivariate analysis) was employed to obtain maximal separation in each pair between the groups, as this technique minimizes the chances of overfitting. Further, we performed Student’s *t*-test with a false discovery rate corrected to determine the statistical significance of signal patterns obtained with *p* < 0.05 in all study groups.

### Pathway and receiver operation characteristic curve analysis

The identified metabolites were employed for mapping pathways. According to pathway topology analysis, each shape has a unique hue and size, depending on its *p* value and pathway impact value respectively. Furthermore, we performed receiver operation characteristic (ROC) curves to determine the metabolites that could distinguish PCOS and control women with high and low BPA levels using MetaboAnalyst 5.0.

### Statistical analysis

The sample size of the study was calculated using Quanto version 1.2.4 (Rudolph et al. 2016). The parameters were set to attain 80% power with the 10% prevalence of the disease and a *p* value of 0.05 to identify 1.5 effect size. The clinical variables were determined by *t*-test, continuous variables were evaluated as mean ± standard deviation, and the correlations between statistically significant metabolites were performed by Pearson’s correlation test using GraphPad prism online tool. Further, statistical significance was considered with a *p* value < 0.05, *p* value < 0.005, and *p* value < 0.0005.

## Results

### Study subjects, clinical parameters, and estimation of urinary BPA levels and their correlation

Based on the study inclusion and exclusion criteria, we recruited 120 PCOS and 119 healthy control females for the study. PCOS women have been found to have higher rates of body mass index, abnormally heavy uterine bleeding, and menstrual cramps than women without the condition (Table 1). In females with PCOS, hyperandrogenism manifests as acne and increased facial and body hair growth in comparison to control females. The modified Ferriman-Gallwey (mFG) scoring system for hirsutism was followed which was significantly higher in PCOS women than healthy controls (*p* < 0.0001). In addition, we observed high level of anti-mullerian hormone in PCOS females than healthy controls. When assessed for urinary BPA levels, we observed significantly elevated levels of BPA in women with PCOS than those of healthy control women (Table 1). Further, the correlation analysis between estimated BPA levels and various clinical variables was performed which revealed an inverse correlation with FSH and positive correlation with Hb1Ac (Table S1).

**Table 1:** Clinical characteristics of the study participants

Demographs	Case (*n* = 120)	Control (*n* = 119)	*p* value
Age (years)	26.6 ± 5.3	31.5 ± 6.4	< 0.0001***
Body mass index (kg/m^2^)	25.2 ± 4.8	23.6 ± 4.4	0.007*
Waist circumference (cm)	92.1 ± 10.7	89.4 ± 14.9	0.108
Marital status (single/married)	37/83	15/104	-
Systolic blood pressure (SBP)	113 ± 9.6	115.9 ± 8.1	0.0017**
Diastolic blood pressure (DBP)	70.4 ± 9.2	73.7 ± 7.5	0.0026**
Glucose profile	Case (*n*)	Control (*n*)	*p* value
Random blood sugar (mg/dl)	103.1 ± 19.3 (60)	101.2 ± 10.8 (43)	0.561
Fasting blood sugar (mg/dl)	94.4 ± 14.7 (37)	104.2 ± 37.2 (17)	0.169
Hb1Ac (%)	5.4 ± 0.6 (40)	5.4 ± 0.9 (45)	1.000
HOMA IR	3.3 ± 1.7 (30)	2.3 ± 1.5 (6)	0.190
Hormone profile and BPA level	Case (*n*)	Control (*n*)	*p* value
TSH (µIU/mL)	3.3 ± 5 (104)	3.2 ± 3 (79)	0.875
FSH (mIU/mL)	6.3 ± 1.8 (45)	7.4 ± 2.2 (15)	0.05
LH (mIU/mL)	8.6 ± 5.6 (44)	7.2 ± 2.5 (13)	0.804
Prolactin (ng/mL)	27.8 ± 29.3 (41)	19.4 ± 7.1 (16)	< 0.264
AMH (ng/mL)	6.0 ± 3.2 (40)	2.2 ± 1.2 (12)	0.0002***
Urinary BPA (μg/g_cr)	4.1 ± 1.3 (120)	3.3 ± 1.2 (119)	< 0.0001***
Clinical hyperandrogenism	Case (*n* = 120)	Control (*n* = 119)	*p* value
Acne (yes/no)	46/74	21/98	-
Alopecia (yes/no)	8/112	0/119	-
Hirsute (Face) (yes/no)	63/57	24/95	-
Hirsute (body) (yes/no)	103/17	93/26	-
Modified Ferriman-Gallwey score (mFG)	6.3 ± 3.6	3.6 ± 1.7	< 0.0001***
Menstrual features	Case (*n* = 120)	Control (*n* = 119)	*p* value
Menarche (years)	13.4 ± 1.4	13.6 ± 1.3	0.254
Menstrual cramps (yes/no)	69/51	44/75	-
Heavy menstrual bleeding (yes/no)	49/71	26/92	-
Infertility status (yes/no/don’t know)	21/40/59	18/73/26	-

All continuous variables are expressed in terms of mean ± standard deviation. Case: PCOS females, Control: healthy control females. *FSH*, follicle−stimulating hormone; *LH*, leutinizing hormone; *AMH*, anti−Mullerian hormone; *TSH*, thyroid−stimulating hormone; *HOMA IR*, homeostasis model assessment–estimated insulin resistance. ***Significant *p* value < 0.0005, **significant *p* value < 0.005, *significant *p* value < 0.05

### Characterization of differential metabolic features among PCOS and healthy females premised on BPA concentrations

After differentiating the plasma samples of the participants into four groups, we further characterized differential metabolites and performed downstream analysis with three target groups (case high, case low, and control high), considering control low as a reference group. From LC–MS data, the features of interest were then selected by performing one-way ANOVA, which yielded 228 significant and 303 unsignificant metabolites (Fig. S1a).

### Partial least square-discriminate analysis

**Fig. 2:**
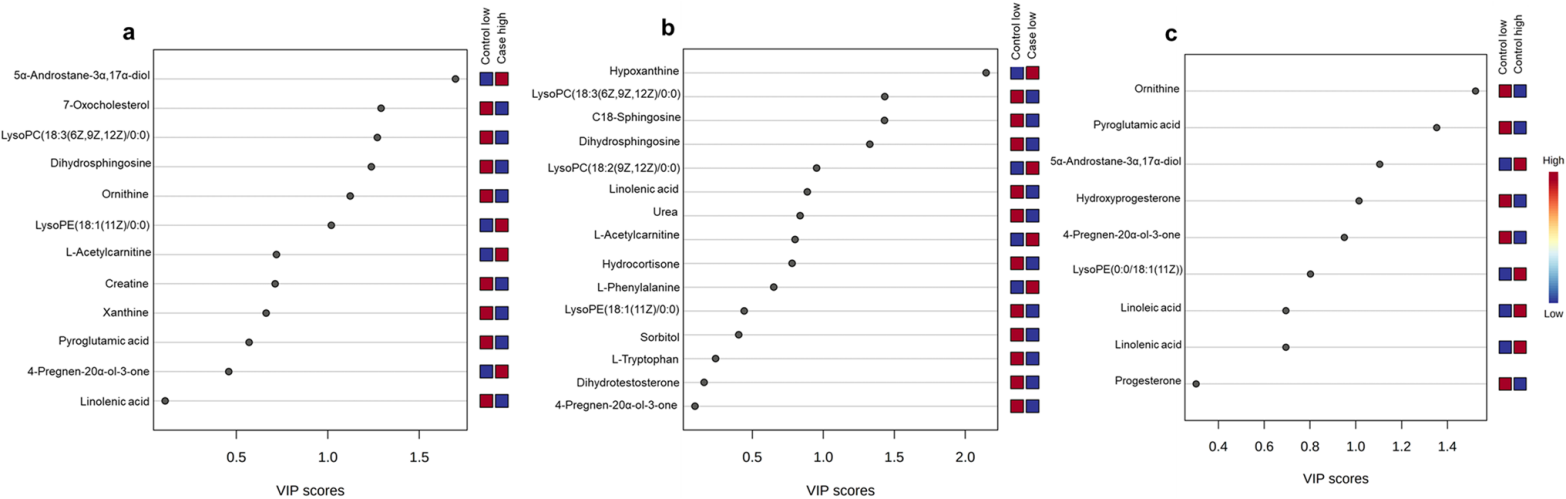
The key features identified by PLS-DA variable importance in projection score of three target groups **a** case high BPA, **b** case low BPA, and **c** control high BPA against control group with low BPA. The colored boxes on the right designate the relative concentrations of the resultant metabolites

The PLS-DA plot certainly illustrates the divergence between the four groups, with components 1 and 2 accounting for 12.2% and 6.5% of the total variance, respectively (Fig. S1b). Among commonly identified metabolites, MS/MS validated 24 features based on their m/z values (Table 2). The key discriminating metabolites that were uncovered by the PLS-DA analysis are displayed in Fig. 2 and sorted in the ascending order of variable importance in projection (VIP) score (Fig. 2). Using a VIP score cut-off of 1.0, 6 features, namely 5α-androstane-3α,17α-diol, 7-oxocholesterol, lysoPC(18:3(6Z,9Z,12Z)/0:0), dihydrosphingosine, ornithine, and lysoPE(18:1/0:0), in the case high group (Fig. 2a), 4 features, namely, hypoxanthine, lysoPC(18:3(6Z,9Z,12Z)/0:0), C18-sphingosine, and dihydrosphingosine, in the case low group (Fig. 2b), and 4 features, namely ornithine, pyroglutamic acid, 5α-androstane-3α,17α-diol, and hydroxyprogesterone, in the control high group (Fig. 2c) were determined to be remarkably strong discriminators between each target group and the control low group. When investigated for common identified metabolites, we found three features, namely, lysoPE(18:1/0:0), pregnenolone, and α-linolenic acid; however, we observed an increasing trend of lysoPE(18:1/0:0) in women with high levels of BPA in comparison to women with low levels of BPA (Fig. S2).

**Table 2:** Catalog of overall detected metabolites validated by LC–MS/MS in PCOS and control women based on their BPA levels

Name	LC–MS m/z	MSMS m/z	Adduct	Fragment present	Total score
Creatine	132.0764	132.0783	[M + H] +	100	86.4
Hypoxanthine	137.0448	137.0457	[M + H] +	80	86.8
Phenylalanine	166.0841	166.0875	[M + H] +	100	82
Tryptophan	205.0941	205.1014	[M + H] +	90.9	84.5
Linoleic acid	281.2429	303.2333	[M + Na] +	100	62.3
Sorbitol	182.0767	220.9346	[M + K] +	100	39.8
Pyroglutamic acid	130.0497	130.0516	[M + H] +	100	82.7
Dihydrosphingosine	302.2995	302.3103	[M + H] +	50	52.7
C18-sphingosine	300.2846	300.296	[M + H] +	56.2	62.7
Dihydrotestosterone	305.2416	305.252	[M + CH3] +	67	49.7
Progesterone	315.2256	315.2264	[M + H] +	47	49.8
L-Acetylcarnitine	204.123	204.1236	[M] +	100	92
Hydrocortisone	363.2113	363.2189	[M + H] +	100	57.1
LysoPC(18:2(9Z,12Z)/0:0)	520.3258	520.3477	[M + H] +	100	83.7
LysoPC(18:3(6Z,9Z,12Z)/0:0)	518.3148	518.3259	[M + H] +	66.7	67.4
LysoPE(18:1/0:0)	480.302	480.3143	[M + H] +	66.7	52.5
Ornithine	133.0974	133.1001	[M + H] +		100
α-Linolenic acid	279.2325	279.2363	[M + H] +	100	63.2
5α-androstane-3α,17α-diol	293.2428	293.2477	[M + H] +		100
Pregnenolone	317.2443	317.252	[M + H] +		99.5
Hydroxyprogesterone	353.1466	353.2043	[M + H] +		99.6
Xanthine	153.0408	153.0429	[M + H] +		99
7-Oxocholesterol	401.3372	401.3393	[M + H] +		100
Urea	61.03992	61.03891	[M + H] +		98.9

### Pathway analysis

**Fig. 3:**
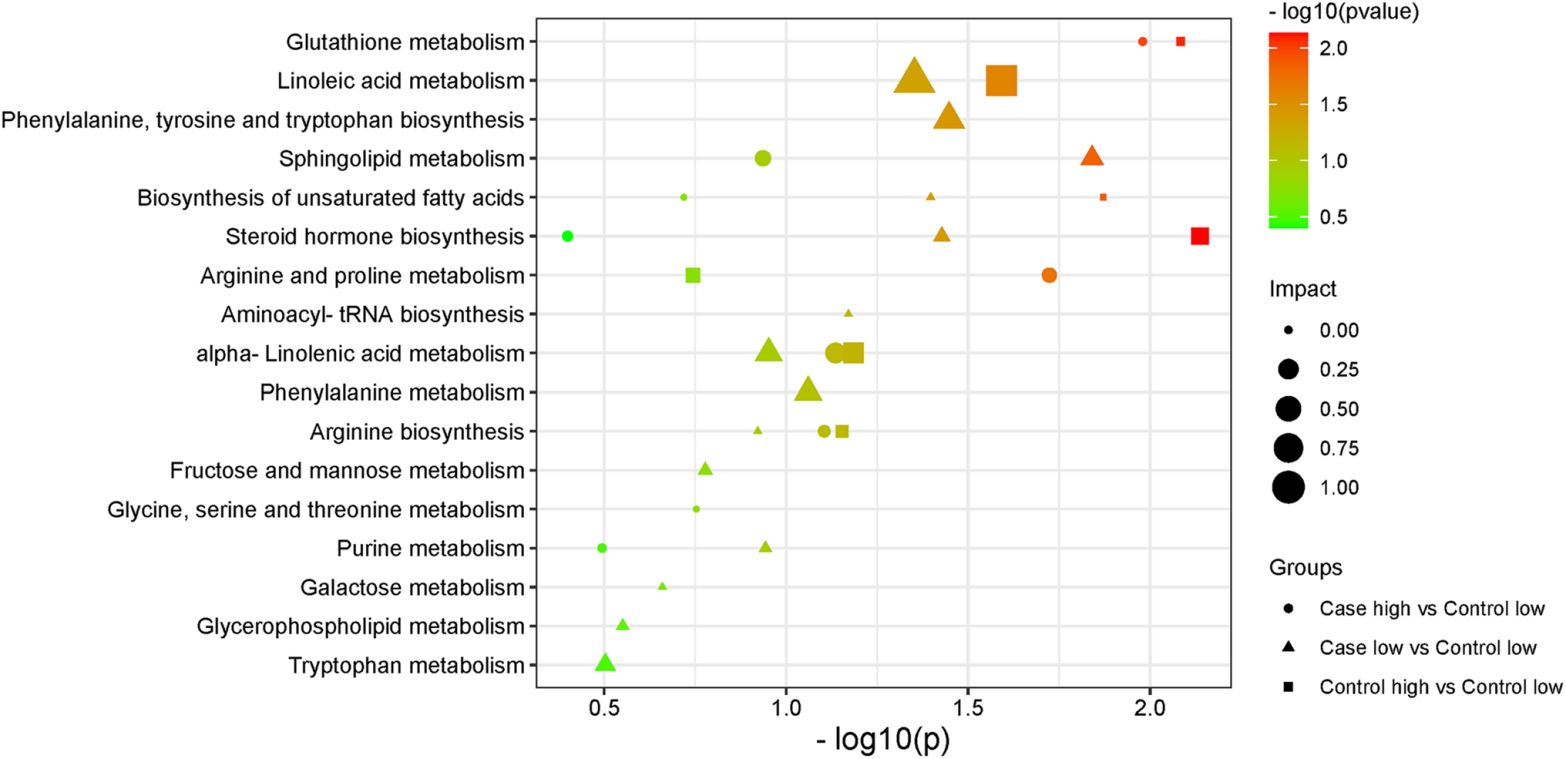
A summary of the global metabolic pathway analysis based on the identified metabolites. On the *y*-axis is the log10 transformed *p* value, while on the *x*-axis is the pathway impact value

The identified metabolites were taken into consideration during the process of determining the disturbed metabolic pathways. The pathway analysis that was based on KEGG showed that the glutathione metabolism, arginine and proline metabolism, α-linolenic acid metabolism, arginine biosynthesis, and sphingolipid metabolism in case high group, sphingolipid metabolism, phenylalanine, tyrosine and tryptophan biosynthesis, linoleic acid metabolism, steroid hormone biosynthesis, phenylalanine metabolism, and α-linolenic acid metabolism in case low, and steroid hormone biosynthesis, glutathione metabolism, linoleic acid metabolism, and biosynthesis of unsaturated fatty acids in control high group were the pathways that were shown to be most affected (Fig. 3).

### Statistical and correlation analysis

**Fig. 4:**
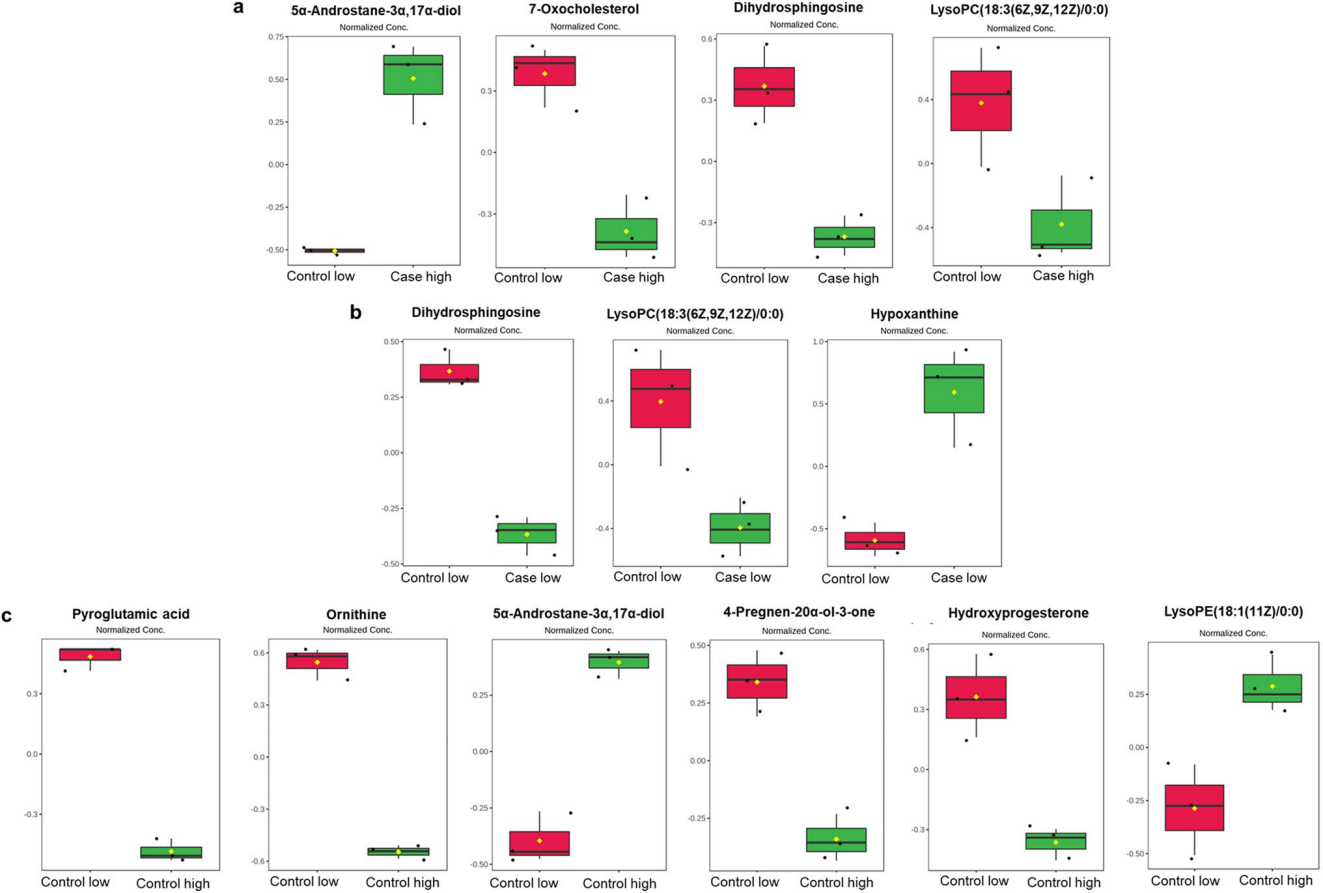
An overview of perturbed metabolites of three target groups **a** case high BPA, **b** case low BPA, and **c** control high BPA against control group with low BPA shown by box and whisker plots

**Fig. 5:**
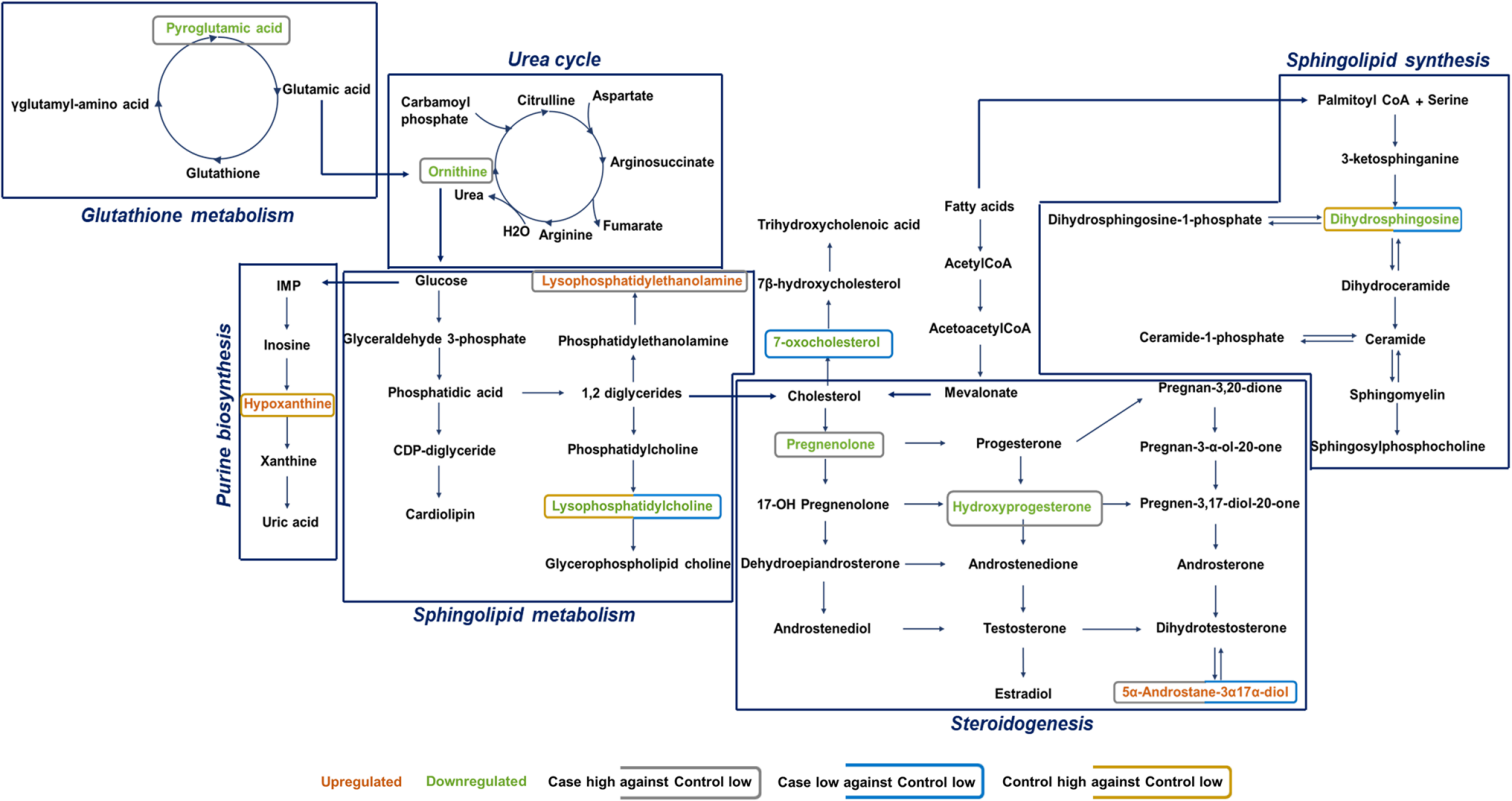
Reconstruction of distorted metabolic pathways

The *t*-test revealed significant differences between certain metabolites that were previously confirmed by VIP score of PLS-DA analysis. 5α-androstane-3α,17α-diol is upregulated and 7-oxocholesterol, lysoPC(18:3(6Z,9Z,12Z)/0:0), and dihydrosphingosine are downregulated in PCOS females having high BPA (Fig. 4a); dihydrosphingosine and lysoPC(18:3(6Z,9Z,12Z)/0:0) are downregulated and hypoxanthine is upregulated in PCOS females having low BPA (Fig. 4b); further, we observed downregulation of ornithine, pyroglutamic acid, pregnenolone, and hydroxyprogesterone and upregulation of 5α-androstane-3α,17α-diol, lysoPE(18:1/0:0) in healthy control females having low BPA (Fig. 4c). Henceforth, these perturbed key metabolites were used for downstream analysis. Further, we reconstructed the metabolic pathway network that highlights the most disturbed processes relying on metabolites that have been considerably altered (Fig. 5). In addition, the key metabolites from each category were evaluated for correlation by Pearson’s correlation test, as depicted in Fig. S3. Here, we identified a significant positive correlation between 5α-androstane-3α,17α-diol and 7-oxocholesterol levels (*r* = 0.63, *p* = 0.03) in PCOS females with high BPA levels and an inverse correlation (*r* =  − 0.62, *p* = 0.01) in healthy control females with low BPA levels (Fig. S3).

### ROC curve analysis

**Fig. 6:**
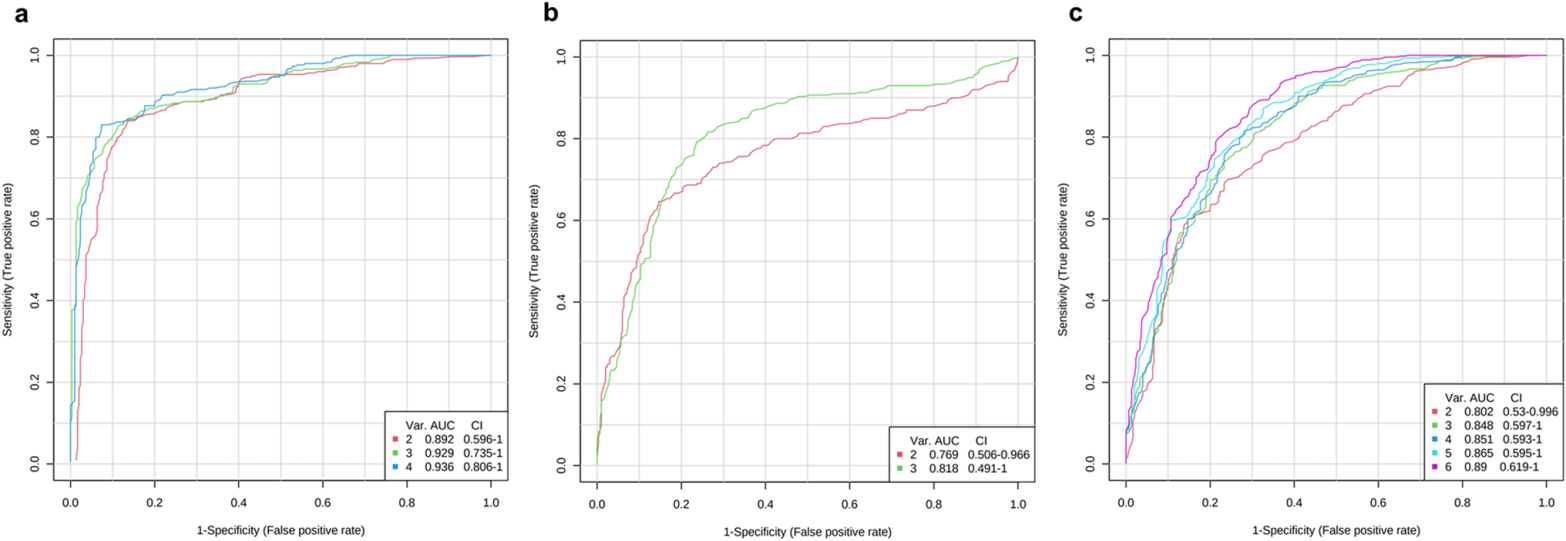
Determining the specificity of identified significant features in distinguishing the three target groups **a** case high BPA, **b** case low BPA, and **c** control high BPA against control group using ROC curves for the metabolite sets

Following that, we carried out a ROC curve analysis in order to determine the diagnostic importance of metabolic biomarkers based on all of the key significant metabolites of the case high, case low, and control high groups against control low group that were obtained. The metabolite set comprising all 4 features of case high, 3 features of case low, and 6 features of control high group with 95% confidence interval showed the maximum prognostic area under the ROC curve (AUC) of 0.936, 0.818, and 0.89 respectively (Fig. 6). The characteristic ROC curves showed the strong discrimination potential, which imply that this model effectively distinguished the plasma samples of the three target groups from the control low group.

## Discussion

BPA among the most prevalent EDC found in daily life has been speculated to be involved in the PCOS pathophysiology by exerting an impact on a wide range of molecular pathways (Kechagias et al. 2020). Numerous human investigations have substantiated the connection between BPA exposure and a wide range of health outcomes, such as perinatal, neonatal, reproductive, and endocrine consequences (Wang et al. 2018). Nevertheless, it has not been determined whether the observed links between elevated BPA levels and PCOS reflect to an etiological aspect or whether the condition’s endocrine signature causes changes in the BPA accumulation and elimination patterns (Kechagias et al. 2020).

Several studies show that BPA levels were considerably higher in women with PCOS compared to normal females and could probably be one of many fundamental origins of this disorder (Kechagias et al. 2020; Kawa et al. 2021). Similarly, our study also revealed elevated urinary BPA levels in PCOS-afflicted women compared to healthy women. Because of the discrepancy between the high levels of BPA found in certain healthy females and the low levels found in some PCOS patients, we set out to investigate the root causes of PCOS in women with low BPA levels and to check whether PCOS-related complications could arise in healthy women with elevated BPA levels as well. As a result, we sought a metabolomics strategy that could generate a plasma metabolic signature which may provide information on the molecular pathophysiology of PCOS induced by BPA.

Considering the inevitability of BPA exposure, molecular markers for earlier detection of the detrimental impact of BPA are essential for enabling the implementation of primary preventative measures (Wang et al. 2018) As in other studies involving Devang et al. (2018) and Ganie et al. (2019), our study also indicated significantly greater BMI and mFG scores, and higher AMH levels in individuals with PCOS than in healthy women (Devang et al. 2018; Ganie et al. 2019). The HbA1c is a reliable marker for insulin resistance (IR). It is well known that PCOS is associated with IR due to its importance in the reproductive process (Adhe Rojekar et al. 2022). The positively significant correlation of Hb1Ac with BPA levels, as revealed by Akash et al. (2022), indicated that individuals with diabetes mellitus were more likely to exhibit IR owing to BPA exposure than non-diabetic individuals (Akash et al. 2022). In keeping with these findings, the correlation analysis between Hb1Ac and BPA levels revealed a positive association in our study, indicating that control females may also be more susceptible to developing IR later in life due to BPA exposure.

Pathway enrichment analysis performed by Tchen et al. (2022) showed steroid hormone biosynthesis as one of the significant altered metabolic pathway linked with BPA exposure (Tchen et al. 2022) indicating a comparable pathway variation in control females of the present investigation. Also as our study indicated, there are certain studies reported that the sphingolipid metabolism, phenylalanine, tyrosine, tryptophan biosynthesis (Yu et al. 2021), and glutathione metabolism (Zhang et al. 2020) were perturbed in PCOS females. Similarly, we also noticed perturbed α-linolenic acid metabolism in women with and without PCOS which may be attributed due to high concentration of urinary BPA. The outcomes of the present study are consistent with existing research findings, despite limited data concerned to metabolic disturbances caused by BPA.

Recent investigations indicate the role of alternate androgen excess pathways in PCOS. Saito et al. (2016) show that the alternate backdoor route in PCOS contributes to excess androgen production (Saito et al. 2016). In women with PCOS, elevated androgen metabolites such as androsterone and androstanediol were produced via both the traditional and alternate backdoor processes. Since these mechanisms of androgen secretion have been overlooked in clinical evaluations of PCOS to date, future research that includes these compounds may benefit in better defining the androgen pattern of PCOS and employing it as a screening tool and reported elevated androstanediol to be the most accurate predictor of PCOS versus controls. In agreement with this, our analyses found elevated androstanediol in PCOS and healthy control individuals having higher BPA than healthy females with lower BPA levels. Further, this pattern in women may be attributed due to high exposure to BPA.

BPA presumably affects *CYP11A1* and inhibits its expression (Peretz et al. 2011). Pregnenolone is necessary for the production of hormones with the estradiol biosynthesis pathway, such as testosterone, androstenedione, progesterone, estradiol, dehydroepiandrosterone, and estrone. Hormone synthesis in antral follicles would be significantly affected by a disturbance in pregnenolone metabolism. Consequently, BPA certainly reduces the *CYP11A1* expression, resulting in a decrease of hormones in the estradiol biosynthesis pathway due to the unavailability of pregnenolone (Peretz and Flaws 2013). In the present investigation, we detected the downregulation of hydroxyprogesterone and pregnenolone in women with elevated BPA levels relative to those with lower BPA levels in controls.

Recent findings have shown that even low-level exposure to BPA can impair metabolic pathways, leading to lipid accumulation in the liver, elevated oxidative stress, and inflammation in females (Cho et al. 2018). Rajska et al. (2020) reported high levels of hypoxanthine and lowered lysophosphatidylcholine (LysoPC) (18:3) concentration in the serum samples of PCOS women (Rajska et al. 2020). Accordingly, our investigation also indicated an elevated hypoxanthine level in low BPA-exposed PCOS females and a diminished level of LysoPC (18:3) in PCOS women compared to healthy individuals. It has been shown that aberrant hypoxanthine levels hinder oocyte meiosis, implying a possible reduction in fertility rate (Hu et al. 2021). Furthermore, reduced lysophosphatidylcholine levels have been connected to obesity and a greater risk of T2DM, both of which exacerbate IR. This could clarify why PCOS individuals are more inclined to develop diabetes. As a result, low LPC concentrations can be a sign of the possibility of PCOS or diabetes in PCOS (Jia et al. 2019) (Vonica et al. 2020).

Regardless of BPA levels, we noticed the downregulation of dihydrosphingosine in PCOS individuals than control ones having low BPA, as reported by Li et al. (2019). Rajska et al. (2020) showed that ornithine is significantly downregulated in PCOS individuals in comparison with healthy females. Researchers also reported the negative influence of BPA on ornithine synthesis in cultured porcine trophectoderm cell lines on exposure of 1 × 10^−4^ M BPA (Elmetwally et al. 2020). When investigating the ornithine levels in the current study, we noticed the downregulation of the metabolite in healthy females with high BPA levels, which reflects the negative impact of BPA among healthy females. A study performed by Cho et al. (2018) indicated that the multiple sphingolipid metabolites were altered due to high exposure of BPA and cause numerous complications in humans. Accordingly, lysoPE(18:1/0:0) was found to be higher in control women with high BPA levels, and a similar pattern of lysoPE was observed in the follicular fluid of the PCOS females revealed by Liu et al. (2019). Hence, we speculate that persistent exposure to BPA in healthy females may have high lysoPE levels and would be more prone to develop PCOS in later stages. A clear inference concerning metabolic perturbations in females with high and low BPA levels requires further investigation. Prior to being utilized in a therapeutic setting, the limitations of the current study should be considered wherein the metabolite profiling was carried out in small sample size hence a validation of the effectiveness of the specified metabolites should be conducted on a larger group and in addition to plasma, urine metabolite profiling also might find more precise indicators of BPA-induced PCOS susceptibility.

## Conclusions

Our findings imply that abnormal metabolite levels offer insight into the potential pathogenic threats linked with BPA exposure in PCOS and healthy females. In addition, this strategy also explored the impact of BPA exposure on disrupted pathways not only in PCOS women, but also in healthy females with elevated BPA levels who were more prone to develop the PCOS phenotype and other associated complications in the future. The utilization of these current findings may aid as a substantial step towards the prediction of PCOS susceptibility induced by BPA and may help in providing better treatment and management strategies for precision medicine.

## Supplementary Information

ESM 1 Differential metabolic outline of PCOS and control women based on their BPA levels. a. Features of interest were chosen using an ANOVA plot with FDR set to a p value cut-off of 0.05. Compounds with their normalized m/z values are displayed along the x-axis, while the log10 of the raw P value within groups is depicted along the y-axis. The FDR significant limit denoted by the dotted lines delineates the boundary between significant and non-significant features. b. Segregation of identified features between 4 groups by 2D PLS-DA score plot of plasma metabolites (PNG 1219 kb)

High resolution image (TIF 6363 kb)

ESM 2 Pattern of plasma metabolite concentration of three target group a. case high BPA b. case low BPA c. control high BPA against control group with low BPA. d. Outline of common metabolites between PCOS and healthy subjects based on their urinary BPA levels. (PNG 746 kb)

High resolution image (TIF 4561 kb)

ESM 3 Pearson's correlation test highlighting altered correlation among metabolites present in three target groups a. case high BPA b. case low BPA c. control high BPA against control group with low BPA (PNG 1211 kb)

High resolution image (TIF 7406 kb)

ESM 4 (DOCX 15 kb)
