# Supplementary figures and images for: Distinct metabolic signatures in blood plasma of bisphenol A–exposed women with polycystic ovarian syndrome
Source: Environ Sci Pollut Res Int. 2023 Apr 15;30(23):64025–35. doi: 10.1007/s11356-023-26820-w (PMC10172238; doi:10.1007/s11356-023-26820-w)

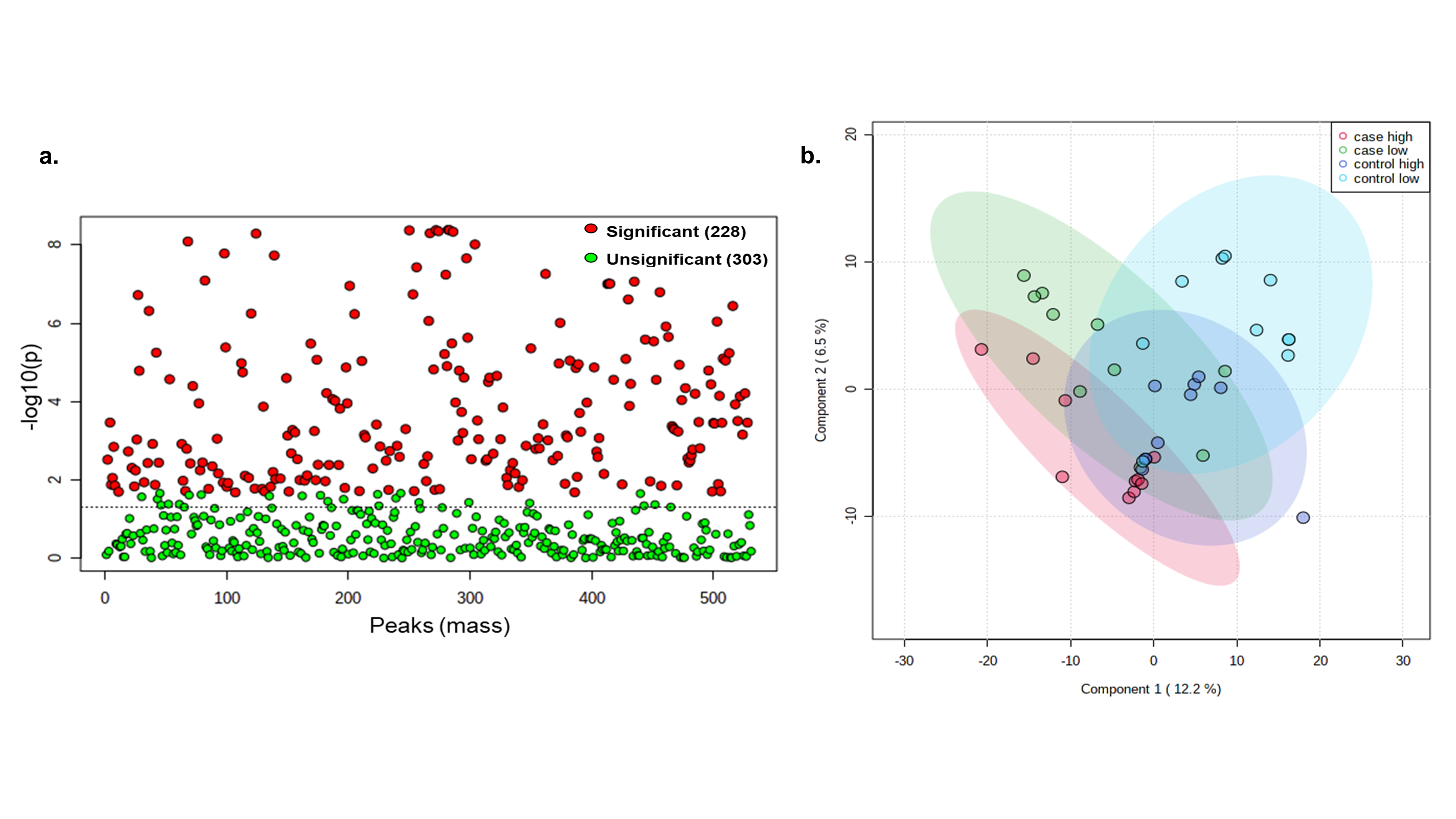

Supplement: Supplementary file 1 — Differential metabolic outline of PCOS and control women based on their BPA levels. a. Features of interest were chosen using an ANOVA plot with FDR set to a p value cut-off of 0.05. Compounds with their normalized m/z values are displayed along the x-axis, while the log10 of the raw P value within groups is depicted along the y-axis. The FDR significant limit denoted by the dotted lines delineates the boundary between significant and non-significant features. b. Segregation of identified features between 4 groups by 2D PLS-DA score plot of plasma metabolites (PNG 1219 kb) [file 11356_2023_26820_Fig7_ESM.png]

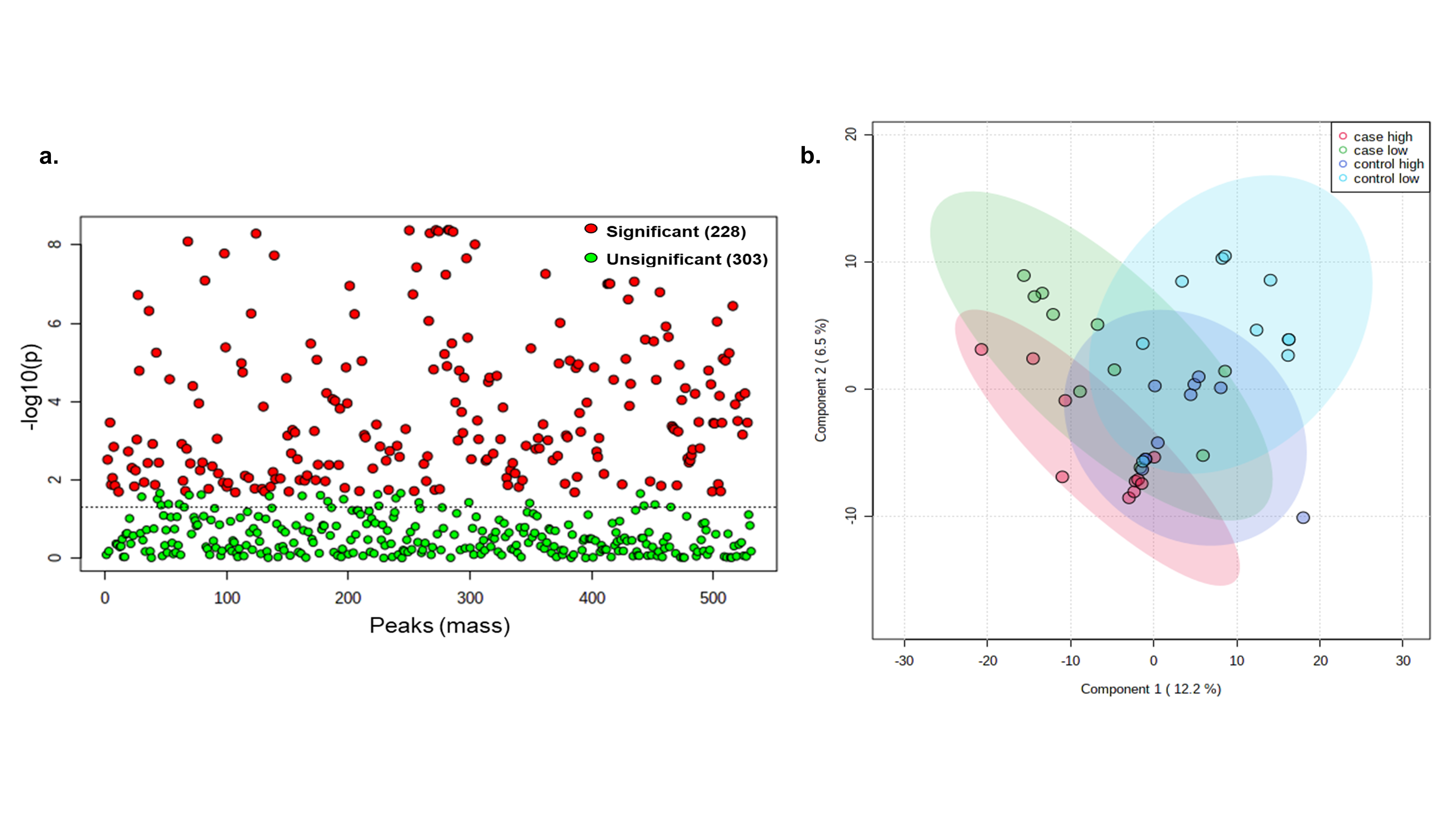

Supplement: Supplementary file 2 — High resolution image (TIF 6363 kb) [file 11356_2023_26820_MOESM1_ESM.tif]

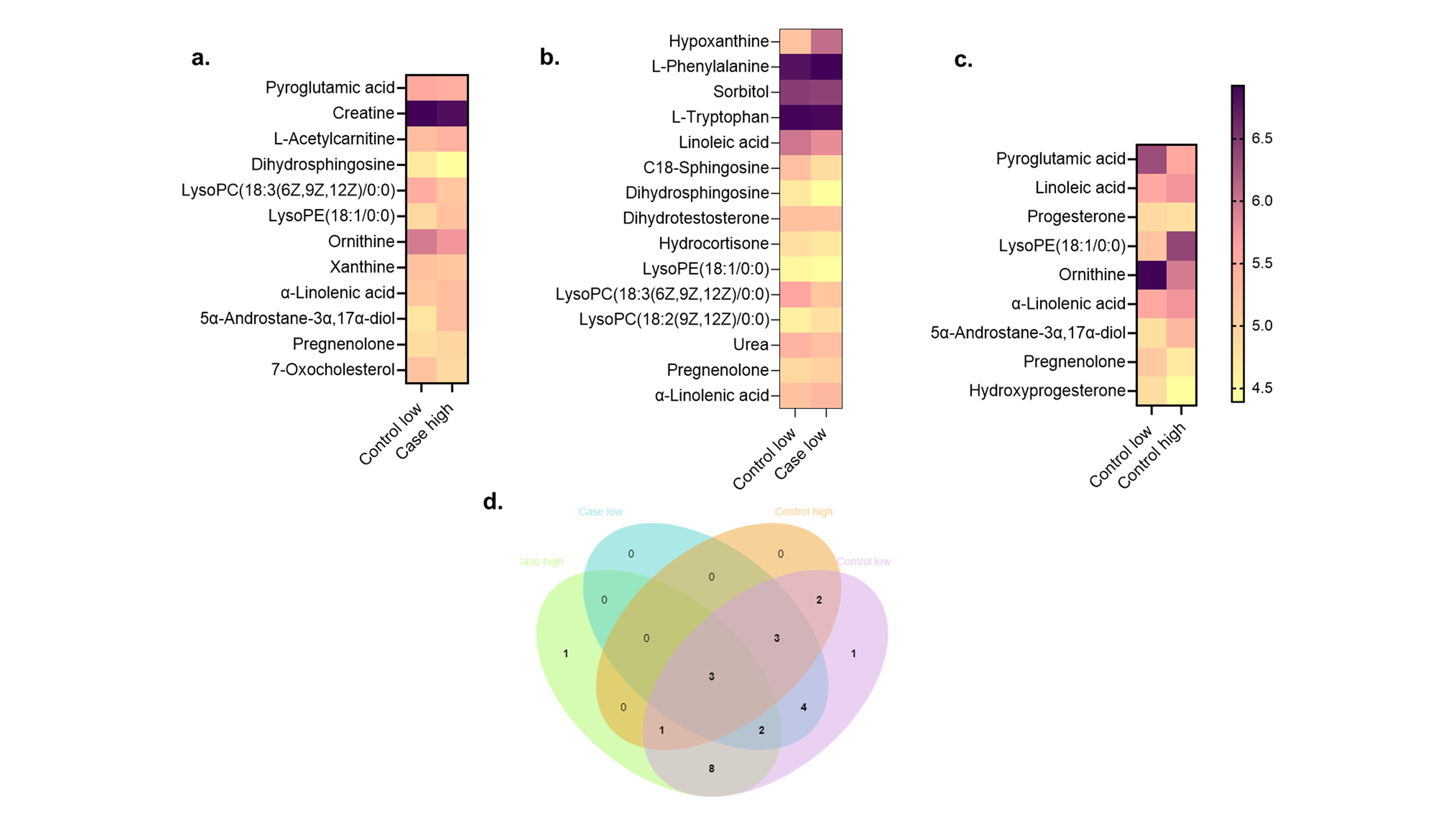

Supplement: Supplementary file 3 — Pattern of plasma metabolite concentration of three target group a. case high BPA b. case low BPA c. control high BPA against control group with low BPA. d. Outline of common metabolites between PCOS and healthy subjects based on their urinary BPA levels. (PNG 746 kb) [file 11356_2023_26820_Fig8_ESM.png]

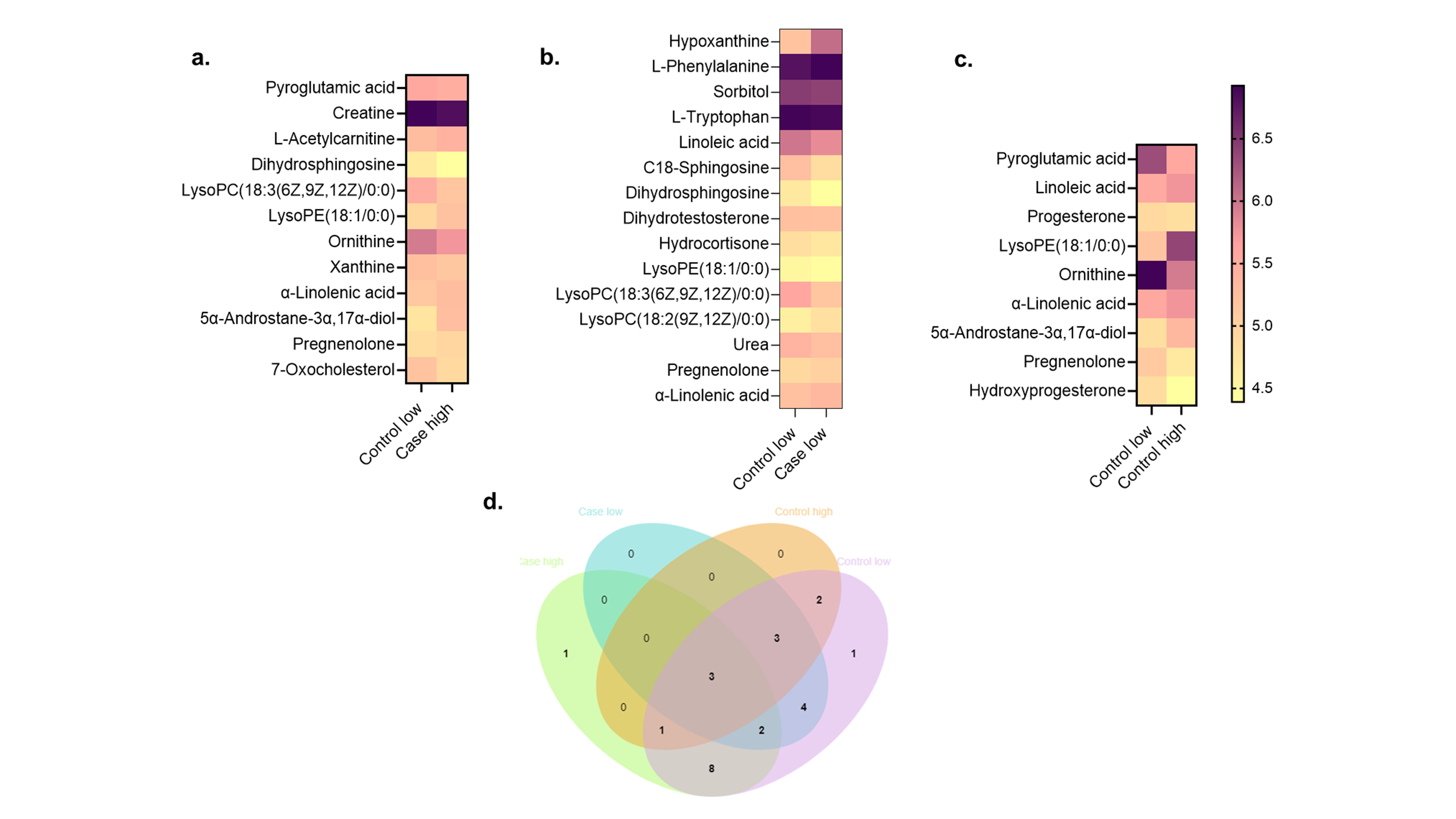

Supplement: Supplementary file 4 — High resolution image (TIF 4561 kb) [file 11356_2023_26820_MOESM2_ESM.tif]

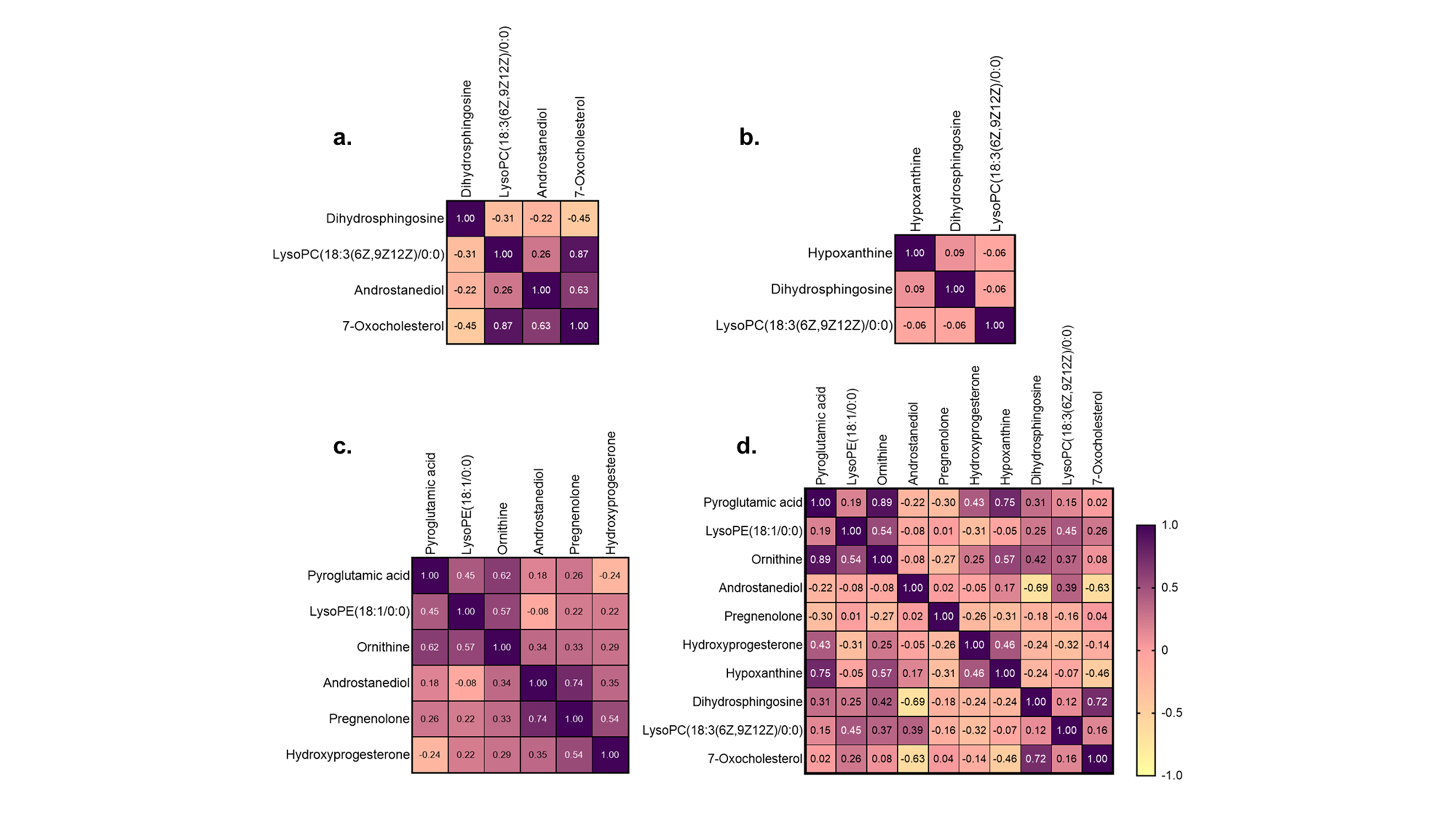

Supplement: Supplementary file 5 — Pearson's correlation test highlighting altered correlation among metabolites present in three target groups a. case high BPA b. case low BPA c. control high BPA against control group with low BPA (PNG 1211 kb) [file 11356_2023_26820_Fig9_ESM.png]

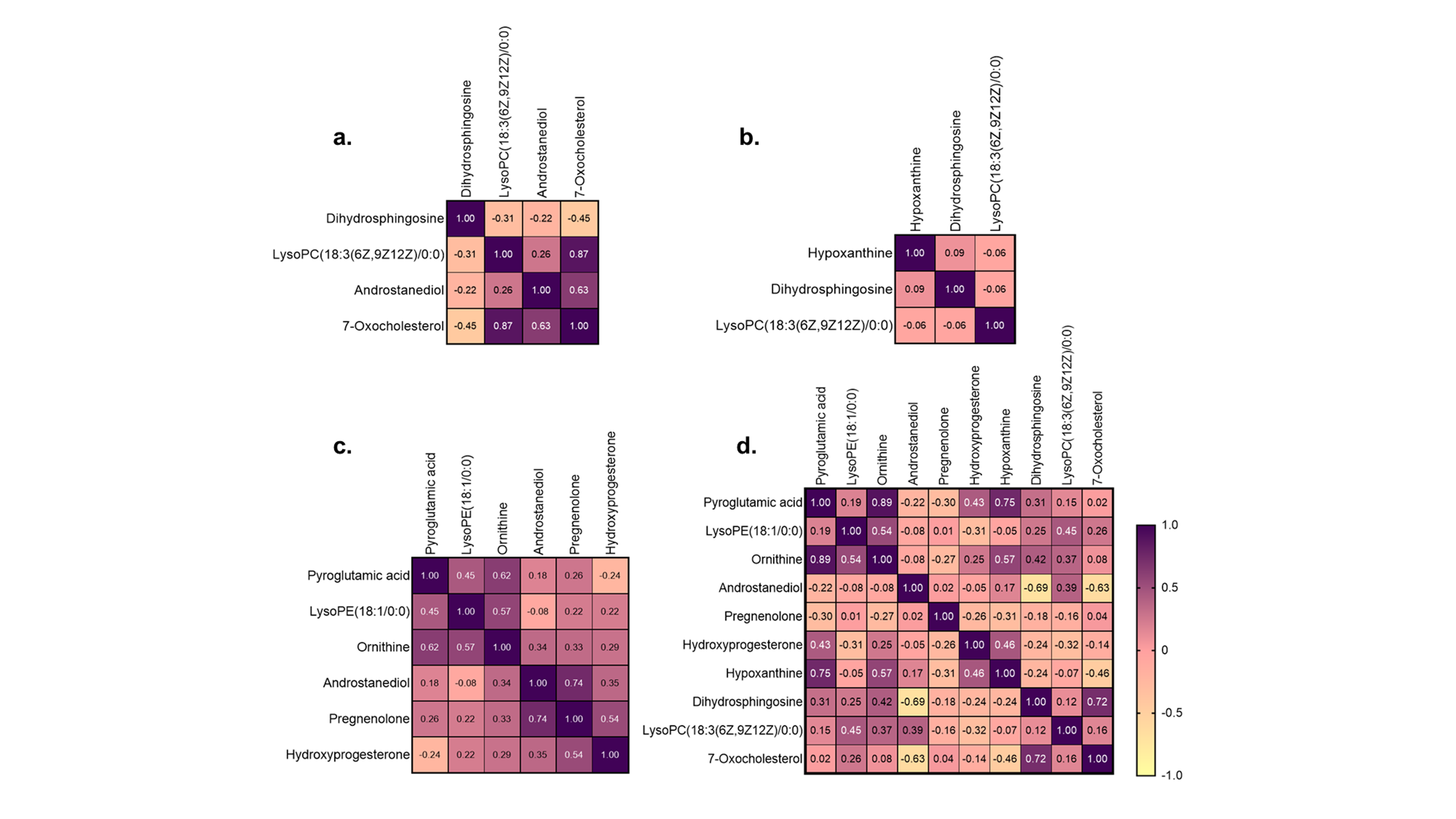

Supplement: Supplementary file 6 — High resolution image (TIF 7406 kb) [file 11356_2023_26820_MOESM3_ESM.tif]
