# Supplementary material for: Distinct metabolic signatures in blood plasma of bisphenol A–exposed women with polycystic ovarian syndrome
Source: Environ Sci Pollut Res Int. 2023 Apr 15;30(23):64025–35. doi: 10.1007/s11356-023-26820-w (PMC10172238; doi:10.1007/s11356-023-26820-w)
Supplement: Supplementary file 7 — (DOCX 15 kb) [file 11356_2023_26820_MOESM4_ESM.docx]

**Table S1** Correlation between BPA levels and clinical parameters

| **Clinical parameters** | **Urinary BPA** | |
| --- | --- | --- |
|  | **PCOS** | **Control** |
| Body mass index (kg/m2) | r(120)= -0.04891, p=0.5958 | r(119)= -0.02670, p=0.7732 |
| Waist circumference (cm) | r(120)= -0.03750, p=0.6843 | r(119)= -0.1207, p=0.1912 |
| Modified Ferriman-Gallwey score (mFG) | r(120)= -0.1511, p=0.0995 | r(119)= -0.09297, p=0.3146 |
| Systolic blood pressure (SBP) | r(120)= -0.04942, p=0.5919 | r(119)= -0.1099, p=0.2342 |
| Diastolic blood pressure (DBP) | r(120)= 0.06915, p=0.4530 | r(119)= -0.07621, p=0.4101 |
| Menarche (Years) | r(120)= -0.01190, p=0.8974 | r(119)= 0.005358, p=0.9539 |
| TSH (µIU/mL) | r(104)= -0.1298, p=0.1891 | r(79)= 0.03470, p=0.7614 |
| FSH (mIU/mL) | r(45)= -0.3874, p=0.0086***** | r(15)= -0.2484, p=0.3720 |
| LH (mIU/mL) | r(44)= -0.004832, p=0.9752 | r(13)= -0.2446, p=0.4206 |
| Prolactin (ng/mL) | r(41)= 0.02579, p=0.8728 | r(16)= -0.1161, p=0.6685 |
| AMH (ng/mL) | r(40)= -0.007071, p=0.9655 | r(12)= -0.4608, p=0.1316 |
| HOMA IR | r(30)= 0.08251, p=0.6647 | r(6)= 0.1407, p=0.7903 |
| Fasting blood sugar (mg/dl) | r(37)= -0.05159, p=0.7617 | r(17)= 0.07573, p=0.7727 |
| Random blood sugar (mg/dl) | r(60)= -0.01491, p=0.9100 | r(44)= 0.1120, p=0.4690 |
| Hb1Ac (%) | r(68)= -0.03721, p=0.7632 | r(46)= 0.3260, p=0.0270***** |

^FSH, Follicle stimulating hormone; LH, Leutinizing hormone; AMH, Anti-Mullerian hormone; TSH, Thyroid stimulating hormone; HOMA IR, Homeostasis Model assessment-estimated Insulin Resistance. * Significant p value < 0.05^
